# Squaring the cube: Towards an operational model of optimal universal health coverage

**DOI:** 10.1016/j.jhealeco.2019.102282

**Published:** 2020-03

**Authors:** Jessica Ochalek, Gerald Manthalu, Peter C. Smith

**Affiliations:** a Centre for Health Economics, University of York, Heslington, York YO10 5DD, United Kingdom; b Department of Planning and Policy Development, Ministry of Health and Population, P. O Box 30377, Lilongwe, Malawi; c Imperial College Business School, Exhibition Road, London SW7 2AZ, United Kingdom

**Keywords:** Priority setting, Coverage, Equity, Global health, Universal health coverage, Cost-effectiveness

## Abstract

Universal Health Coverage (UHC) has become a key goal of health policy in many developing countries. However, implementing UHC poses tough policy choices about: what treatments to provide (the depth of coverage); to what proportion of the population (the breadth of coverage); at what price to patients (the height of coverage). This paper uses a theoretical mathematical programming model to derive analytically the optimal balance between the range of services provided and the proportion of the population covered under UHC, using the general principles of cost-effectiveness analysis. In contrast to most CEA, the model allows for variations in both the costs of provision and the social benefits of treatments, depending on the deprivation level of the population. We illustrate empirically the optimal trade-off between the size of the benefits package and the proportion of the population securing access to each treatment for a hypothetical East African country, based on WHO data on the costs and benefits of treatments at different coverage levels. We begin with a scenario allowing coverage levels to vary, then apply differential equity weights to the benefits of coverage, and finally illustrate a scenario where interventions are either provided at 95% coverage or not at all (as is usually done in health benefits package design) for comparison. The results present the optimal trade-off between the social benefits of pursuing full population coverage, at the expense of expanding the benefits package for ‘easier to reach’ populations.

## Introduction

In many low- and middle-income countries (LMICs) recent debates about health policy have focused on the notion of Universal Health Coverage (UHC). This is defined by the World Health Organization as “ensuring that all people can use the promotive, preventive, curative, rehabilitative and palliative health services they need, of sufficient quality to be effective, while also ensuring that the use of these services does not expose the user to financial hardship” (WHO, 2018). UHC was the topic of the 2010 World Health Report, and the subject was given further impetus by the adoption in 2012 of a United Nations General Assembly Resolution on UHC, and its inclusion as the central health-related feature of the Sustainable Development Goals (United Nations, 2013; WHO, 2010, 2017).

As explained by Glassman et al. (2017), the attraction of UHC to policymakers is easy to understand. In principle, it improves access to health services for many people who would otherwise be unable to use those services, reduces the incidence of serious impoverishment caused by health shocks, and, by making access to health services unrelated to ability to pay, promotes a widely held concept of fairness. Furthermore, it has been shown to be an efficient way of improving health outcomes for the population (Moreno-Serra and Smith, 2015). Most high-income countries (HICs) have had some form of UHC in place for several decades, and an increasing number of LMICs are seeking to make a transition towards UHC.

A fundamental principle of UHC is that it should be funded by government or quasi-government sources (such as mandatory social health insurance or donor funds) (Nicholson et al., 2015). In most settings the bulk of this funding will arise from taxes or insurance payments by individual citizens, which are then pooled for the purposes of UHC. The important characteristic of whatever funding mechanism is used is that contributions should be unrelated to medical risk, as indicated for example by the health status or age of the citizen. In order for the funding pool to be sustainable, it will also usually be the case that contributions should be mandatory. Of course, the UHC funding pool can be augmented by other sources, such as donor funds or corporate taxes. We do not discuss further the nature of the funding pool, but for this paper assume it is exogenously fixed for a given period by the government or an analogous national decision-making body.

**Fig. 1:**
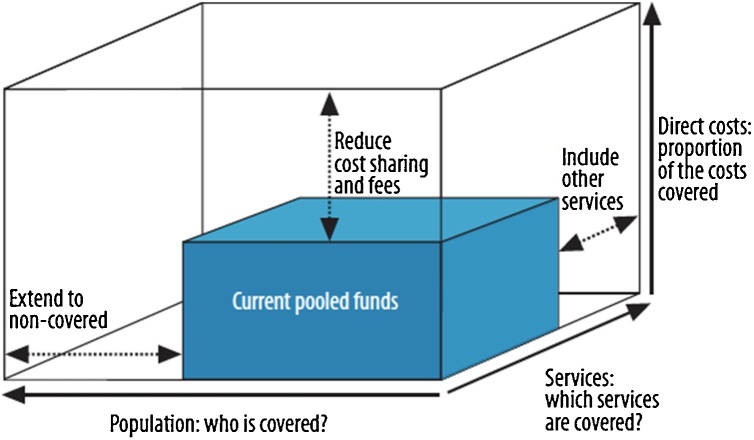
Three dimensions to consider when moving towards UHC. Source: (WHO, 2015).

Any system of UHC is therefore constrained in scope by the funds available, from whatever source, which in LMICs are vastly inferior to those available in HICs. Whilst limited resources do not usually compromise the principle of UHC, they expose policymakers to some particularly tough policy choices that have largely been finessed in higher income countries. Specifically, in implementing UHC, policymakers must seek out the best use of limited funds, whilst respecting the principles of UHC. The WHO has characterized the policy problem as a ‘cube’, the size of which indicates the resources available for UHC (Fig. 1). (Strictly speaking, because its sides are not equal, the ‘cube’ should be referred to as a rectangular cuboid, but that nomenclature lacks the rhetorical impact of ‘cube’!). Using this device, it is argued that the core of the decision problem requires decisions along three dimensions:

- What treatments should be included in the defined package of benefits (sometimes referred to as the depth of coverage)?
- What part of the population should enjoy access to the treatments (the breadth of coverage)?
- What charges should patients incur for using the UHC treatments (the height of coverage)?

Using this representation, the UHC policy problem can be seen as a constrained optimization problem, in which some concept of social welfare must be maximized subject to the overall budget constraint represented by the cube. The decision variables are indicated by the three broad dimensions of the cube. To date, most analysis and debate has focused on optimizing the depth of coverage (the range of treatments to which beneficiaries are entitled) (Glassman et al., 2017). The creation of such a health benefits package is a fundamental requirement for effective implementation of UHC. However, UHC policy should be viewed alongside the other two dimensions of the cube. As a first step, we offer an approach towards informing policy on the breadth of the population to be covered by UHC, as well as the range of services.

Jamison et al. (2013) have argued for fully covering a limited range of treatments, particularly targeted toward the poor. However, in practice, most implementation of UHC has failed to secure 100% of population coverage for many of the treatments that are purportedly within the UHC benefits package (Nicholson et al., 2015; Somanathan et al., 2014). This shortfall in access leaves funds available to increase the range of services covered in the benefits package for those who enjoy access, but is usually in contradiction to the objective of securing ‘universal’ health coverage for the whole population. In short, ‘depth’ of coverage is increased at the expense of ‘breadth’. This trade-off lies at the heart of this paper. When the policy intention is in principle to secure 100% access to the chosen treatments, any shortfall can be thought of as being ‘unmet need’. It may occur for a range of reasons, such as the direct and indirect financial costs to the individual of securing access, cultural and informational barriers to access, or simply a failure to provide the service. It must be emphasized that a shortfall in access will not in general arise from deliberate policy to refuse treatment to certain groups. Rather, it occurs because of such indirect influences on access. In this paper, we assume that such barriers to access can be removed, albeit at a cost to the health system, for example in the form of improved transport provision or introduction of health facilities in remote rural areas.

Some countries seeking to implement UHC impose charges on some or all of the population for using certain treatments in the UHC benefits package. Of course, if the charge is as high as the prevailing market price for a treatment, it is effectively removed from the benefits package. However, assuming the user charge is less than the full market price, the treatment is partially subsidized and can therefore be considered to be within the benefits package. User fees increase the total funds available for UHC by (a) reducing demand for the affected treatments and (b) directly yielding income from patients for the health system. However, they can compromise a fundamental principle of UHC by exposing the user to financial hardship, and therefore diluting financial protection (Smith, 2005). For completeness, we make reference to user charges in the theoretical model set out below. However, because of the complexity of specifying and enforcing user charges in many LMIC settings, they are often not considered a viable policy option, and so we do not incorporate them into our core analysis.

The WHO cube (first proposed by Reinhard Busse and colleagues (Busse and Schlette, 2007)) has proved an immensely helpful device for highlighting to policymakers the essential trade-offs that are made when seeking to make a transition towards UHC (or improve the effectiveness of an existing implementation). However, it is a conceptual device, and not a practical tool for detailed design of UHC. It makes no pretense to modelling the immense diversity of potential treatments that might be included in a health benefits package, or the variations in access secured by the population entitled to use those treatments.

In practice, no system of UHC has been able to respect all the principles embodied in UHC. For example:

- It has rarely been possible to specify fully an explicit set of treatments to which all beneficiaries are entitled to use (and, by implication, a complementary set of treatments that are excluded from the benefits package);
- It is rare to find that 100% of the population actually secures access to all UHC services to which they are entitled. Instead, to a greater or lesser extent, all systems of UHC exhibit aspects of ‘unmet need’, which may arise from financial, geographical, cultural or informational barriers to access;
- Even within comprehensive systems of UHC, there is considerable evidence of financial hardship caused by using health services. Several measures of ‘catastrophic’ and ‘impoverishing’ spending on health services have been developed (Boerma et al., 2014; Wagstaff and Doorslaer, 2003; World Health Organization Regional Office for Europe, 2017), although these generally are not able to distinguish between spending on UHC services and spending on other services, or to capture the indirect costs to patients of securing access, such as transport costs;
- There are numerous examples of inefficiency in all health systems (which the WHO estimate to be between 25% and 30% of all spending (WHO, 2010)), effectively reducing the size of the cube available for UHC;
- The treatments delivered to patients may not always be delivered to the ‘acceptable’ level of quality referred to in the WHO definition of UHC. This has led to development by WHO of the concept of ‘effective’ coverage, referring only to that proportion of the population receiving service of acceptable quality (Ng et al., 2014).

A variety of analytic techniques have been developed to offer policy guidance on the effectiveness of UHC, such as developing a health benefits package, measuring levels of effective coverage, promoting financial protection, and improving the efficiency of services. However, these have in general been developed in a piecemeal fashion and not been incorporated into a general model of UHC optimization.

The purpose of this paper is to offer a first step in the development of a general model for optimizing the use of UHC funds by focusing on two of the three dimensions of UHC – the trade-off between breadth and depth of coverage. In the next section we develop a rudimentary theoretical model of UHC optimization. Based on the theoretical model, we then make use of available data to provide an illustration of our operational approach towards the optimal design of the discretionary part of a UHC benefits package. We then describe the data and methods used, followed by a discussion of the results of the illustration and their implications for understanding the trade-off between the depth and breadth of coverage. We conclude with a discussion of the limitations of this approach and the opportunities offered.

## A theoretical model of UHC

The approach adopted in this paper, reflecting the formulation of UHC as a ‘cube’, is to represent the UHC design problem as a constrained optimization. In its most general form, we assume UHC seeks to maximize some concept of social welfare subject to a fixed health system budget constraint. A general theoretical approach towards securing an optimal trade-off between the three dimensions of coverage is set out in Appendix 1, adapting a framework suggested by Smith (2005, 2013). In this section, we abstract the elements of the model relevant to modelling the trade-off between the breadth and depth of coverage.

We assume that the population is heterogeneous in some level of disadvantage *y*, distributed according to a density functionγ(y). For expository reasons, we sometimes refer to *y* as ‘wealth’. However, particularly in LMICs, disadvantage may extend beyond conventional measures of wealth, to include factors such as remoteness and educational opportunities. The precise definition of *y* is not material to model. We then express individual utility as an increasing function of both health *h* and wealth *y*, and assume that social welfare is some (possibly weighted) aggregation of individual utility. Our general formulation (set out fully in the appendix) then seeks to capture (a) the health improvement aspects of UHC; (b) the financial protection function of UHC; and (c) any additional equity objectives of UHC (beyond the redistributive mechanism implicit in its design). We assume there is a set of *N* efficient health treatments *i*, each of which addresses a discrete health problem, and creates a health benefit for the individual of *b_i_*, which may be expressed in the form of quality-adjusted life years (QALYs) or their disability adjusted life year (DALY) counterparts. In the first instance our theory assumes that *b_i_* is constant at all levels of *y*, but this can readily be relaxed (see below). The costs of supplying the treatment *x_i_(y)* are assumed to be non-increasing in *y*, reflecting the additional costs often associated with delivering effective treatments to disadvantaged groups$x_{i}^{'}(y)\leq 0$. We also allow for the potential in the theoretical model for variable epidemiology of each disease by allowing the incidence – the probability of needing treatment $\pi_{i}(y)$ - to vary with disadvantage. Due to data constraints, in our illustration below, estimates of costs and benefits are, however, treated as a function of coverage level rather than wealth. Throughout we assume a single time period (although of course the benefits of treatment may extend well beyond that period) and a fixed annual government budget *X*.

On the demand side we adopt a simple concept of individual utility that is linear in health (as is implicit in traditional cost-effectiveness analysis) and is separable in *h* and *y*, such that *u(h,y) = h + v(y)*, where *h* is the individual’s level of health and *y* her disadvantage. We assume *v (.)* has the usual properties $v^{'}(.)\geq 0$ and$v^{''}(.)\leq 0$. When a health shock arises, the individual is assumed to accept treatment if the value of the health benefits of treatment *b_i_* exceeds the impact of its price to the individual *p_i_*, that is $b_{i}\geq v\left(y\right)-v\left(y-p_{i}\right)$. Conversely, treatment is foregone if the health benefits do not compensate for the impact of the price of access, that is $b_{i}\leq v\left(y\right)-v\left(y-p_{i}\right)$. In the formulation adopted in this paper, we assume zero price for all individuals, although we at first retain the notion of a price to maintain consistency with the UHC cube.

In order to reflect equity concerns, a policy weight *w(y)* can be attached to a person at each level of disadvantage *y*, with the assumption that this is skewed in favour of disadvantaged populations, $\;w_{i}^{'}(y)\leq 0$. Note that the use of *w (.)* is equivalent to relaxing the assumption that the health gains of treatment are equal across all individuals. This may be the case, for example, if more deprived populations have access to fewer alternative treatments, rendering the treatment under consideration more valuable than to their less deprived counterparts. We then assume that the social welfare function comprises the (weighted) sum of individual utility losses associated with a set of payments *p_i_*:

$$SWL=\sum_{i}\left\{\int_{0}^{\psi \left(p_{i}\right)}b_{i}w(y)\pi_{i}\left(y\right)\gamma \left(y\right)dy+\int_{\psi \left(p_{i}\right)}^{\infty }\left[v\left(y\right)-v(y-p_{i})\right]w(y)\pi_{i}\left(y\right)\gamma \left(y\right)dy\right\}$$

where for each treatment there is a critical level of wealth $\psi (p_{i})$ at which there is indifference between treatment and no treatment with price *p_i_*. The first term in the expression is the expected health loss of those who do not secure access to care, and the second reflects the financial loss of those who do use care.

The costs of providing treatment (net of any user fee income) are constrained by the availability of public funds *X*:

$$\sum_{i}\int_{\psi \left(p_{i}\right)}^{\infty }\left[x_{i}(y)-p_{i})\right]\pi_{i}\left(y\right)\gamma \left(y\right)dy\leq X$$

There is in addition a constraint on the value each price *p_i_* can take, which is no greater than the market price *M_i_* so that $0\leq p_{i}\leq M_{i}$. The implications for optimizing this full model of UHC are set out in the appendix.

In this paper we consider the case in which no user fees can be charged. Then the policy problem is the trade-off between maximizing coverage and maximizing aggregate health gain, according to the chosen social welfare function. In this case, the absence of user fees implies reformulating the problem as:

$$\text{M}\text{a}\text{x}\text{i}\text{m}\text{i}\text{z}\text{e}\underset{i}{\sum }\overset{\infty }{\underset{z_{i}}{\int }}b_{i}w(y)\pi_{i}\left(y\right)\gamma \left(y\right)dy\;\;\;\text{s}\text{u}\text{b}\text{j}\text{e}\text{c}\text{t}\;\;\text{ t}\text{o}\;\;\;\underset{i}{\sum }\overset{\infty }{\underset{z_{i}}{\int }}x_{i}(y)\pi_{i}\left(y\right)\gamma \left(y\right)dy\leq X$$

This expression maximizes weighted health gains subject to the budget constraint. The decision variables $z_{i}$ indicate the level of wealth below which treatment *i* cannot be offered (because the benefits of reaching the disadvantaged populations are outweighed by the opportunity costs to the rest of the health system). For interior solutions this leads to first order conditions for each treatment *i*:

$$b_{i}w\left(z_{i}^{*}\right)\pi_{i}\left(z_{i}^{*}\right)\gamma \left(z_{i}^{*}\right)=\lambda x_{i}\left(z_{i}^{*}\right)\pi_{i}\left(z_{i}^{*}\right)\gamma \left(z_{i}^{*}\right)$$

where λ is the Lagrange multiplier. This can be rewritten $b_{i}w\left(z_{i}^{*}\right)/x_{i}\left(z_{i}^{*}\right)=\lambda$, indicating that a critical level of wealth (and therefore coverage) for each treatment is determined by the ratio of (equity weighted) benefits to production costs. This is the standard cost-effectiveness rule, except that costs and (weighted) benefits are allowed to vary with disadvantage. At the margin, the benefit of further extending coverage (additional health gain, possibly weighted for equity) is just offset by the marginal cost to the health system of such extension.

In the absence of equity weights *w(.)*, the results are straightforward to interpret, assuming costs increase monotonically with disadvantage. If the entire population is covered $z_{i}^{*}=0$, then the condition becomes $b_{i}w\left(0\right)/x_{i}\left(0\right)\geq \lambda$. When none are covered by publicly funded services, only those who are prepared to pay the market rate *M_i_* will secure access, then $b_{i}w\left(z_{i}^{*}\right)/x_{i}\left(z_{i}^{*}\right)\leq \lambda$.

The introduction of equity weights (or equivalently non-constant health benefits) renders analytic first order conditions unhelpful from a practical point of view, as there may be multiple local solutions to the optimization, and therefore more than one value of z_i_* satisfying the first order condition. For example, the concern with equity may be so great that provision may be optimal for some very disadvantaged (high cost) groups, and also some low cost groups, but not for the entire population. In most circumstances this ‘care gap’ in service provision between (say) very rich and very poor people is politically and practically infeasible. We therefore require that only one cut-off wealth level can be applied. This may mean that – for some groups – the costs of provision exceed the benefits at an optimal solution, as measured by a conventional cost-effectiveness criterion, but this loss is outweighed by the benefits of extending provision of services to the most disadvantaged, after equity concerns are taken into account.

This constrained solution concept for each treatment is equivalent to seeking out the single level of disadvantage $z_{i}^{*}$ at which the ‘net health benefit’ of implementing the treatment is maximized for a given λ, subject to all citizens in the range $\left[z_{i}^{*},\infty \right]$ being covered, and none in the range $\left[{0,z}_{i}^{*}\right)$. Analytically, this requires identifying for each treatment *i*:

$$\text{M}\text{a}\text{x}\text{i}\text{m}\text{i}\text{z}\text{e}\;\;\;\overset{\infty }{\underset{z_{i}}{\int }}b_{i}w(y)\pi_{i}\left(y\right)\gamma \left(y\right)dy-\;\overset{\infty }{\underset{z_{i}}{\int }}{\lambda x}_{i}(y)\pi_{i}\left(y\right)\gamma \left(y\right)dy$$

This expression indicates the weighted health benefits of implementing the treatment at coverage level *z_i_* less the health opportunity costs of implementation (calculated with reference to the Lagrange multiplier λ from the initial optimization). If net health benefits are positive for some levels of disadvantage, the treatment should be implemented at the coverage level $z_{i}^{*}$ that maximizes those benefits. Of course, negative values of the expression at all levels of *y* imply that the treatment should not be provided at all under UHC. Implementing this principle requires an iterative approach to determining the optimal level of λ, as described below.

## Data

Implementation of the model requires data on the costs and benefits for each intervention and coverage level under consideration and the size of the patient population that stands to benefit from each intervention. While data on the costs and benefits of implementing an intervention for the full population in need tends to be readily available from cost-effectiveness studies, costs and benefits generally assume full coverage and are rarely broken down by coverage level. One exception is region-level data published by WHO-CHOICE for a limited range of treatments at three different levels of coverage (50%, 80% and 95%) (Tan-Torres Edejer et al., 2003).

We use WHO-CHOICE data for 16 interventions published for the AFR-E region^1^: community newborn care package, tetanus toxoid, screening and treatment of syphilis, normal delivery by a skilled attendant, management of maternal sepsis, management of serious newborn infections (Adam et al., 2005; WHO, 2014d); insecticide-treated bed nets (ITN), intermittent presumptive treatment in pregnancy (IPTP), case management with artemisinin-based combination therapy (ACT) (Morel et al., 2005; WHO, 2014b); measles rubella vaccine, vitamin A supplementation in pregnant women, management of severe malnutrition (children), vitamin A supplementation in infants and children 6–59 months, pneumonia treatment (children), zinc (Edejer et al., 2005; WHO, 2014a); and treatment of new smear-positive TB cases only under DOTS (Baltussen et al., 2005; WHO, 2014c).

The WHO-CHOICE data gives total costs and benefits (measured in DALYs) at three levels of coverage: 50%, 80% and 95%. We assume constant incremental costs and benefits up to 50% coverage. We then infer incremental unit costs and benefits at additional coverage levels (55%, 60%, 65%, 70%, 75%, 80%, 85%, 90%, 95% and 100%) by interpolation. Because WHO-CHOICE provide three data points we are able to assess whether linearity is assumed, and where it is not, we fit a quadratic relationship from which costs at intermediate coverage levels can be calculated. (Data and details are available in the Appendix 2.) These approximated relationships are differentiated with respect to coverage to obtain the marginal costs (2000 Int$) and marginal benefits at 50%, 55%, 60%, 65%, 70%, 75%, 80%, 85%, 90%, 95% and 100% coverage. We then convert the costs into 2015 US$ for the AFR-E region.

In order to calculate budget impact, data on the annual incidence of disease is required. We use data from the Global Burden of Disease and Demographic and Health Surveys Stat Compiler tool for 2015 for a representative country in the AFR-E region.^2^ Data are summarized in Table 1, which presents the costs and benefits by 50%, 80% and 95% coverage and the annual incidence for a population of 25 million.

**Table 1:** WHO–CHOICE costs and benefits by coverage level and patient population.

	Cost per year (I$, millions) per capita			DALYs averted per year per capita			Incident population (2015)	
Intervention	50%	80%	95%	50%	80%	95%	Number	Definition
Community newborn care package	0.089	0.145	0.179	0.011	0.017	0.020	27,662	Population 0–27 days of age
Tetanus toxoid	0.058	0.118	0.194	0.005	0.007	0.009	1,067,218	Population <1 year
Screening and treatment of syphilis	0.034	0.070	0.119	0.000	0.001	0.001	595,029	Percentage of women 15–49 currently pregnant * population female 15–49
Normal delivery by a skilled attendant	0.157	0.259	0.334	0.004	0.007	0.008	595,029	Percentage of women 15–49 currently pregnant * population female 15–49
Management of maternal sepsis	0.088	0.154	0.219	0.001	0.001	0.002	70,148	Incidence of maternal sepsis and other maternal infections
Management of serious newborn infections	0.150	0.268	0.403	0.003	0.004	0.005	11,264	Incidence of neonatal sepsis and other neonatal infections
Measles rubella vaccine	0.100	0.162	0.224	0.001	0.003	0.003	1,067,218	Population <1 year
Insecticide-treated bed nets (ITN)	0.474	0.629	0.710	0.010	0.015	0.017	595,029	Percentage of women 15–49 currently pregnant * population female 15–49
Intermittent presumptive treatment in pregnancy (IPTP)	0.054	0.057	0.060	0.000	0.000	0.000	595,029	Percentage of women 15–49 currently pregnant * population female 15–49
Case management of malaria with artemisinin-based combination therapy (ACT)	0.192	0.203	0.211	0.009	0.015	0.017	8,375,236	Incidence of malaria
Treatment of new smear-positive TB cases only under DOTS	0.428	0.768	1.069	0.068	0.109	0.130	112,918	Incidence of TB
Vitamin A supplementation in pregnant women	0.077	0.394	0.725	0.001	0.002	0.003	595,029	Percentage of women 15–49 currently pregnant * population female 15–49
Management of severe malnutrition (children)	5.039	8.085	9.651	0.000	0.000	0.000	122,555	Incidence of protein-energy malnutrition among children under-5
Vitamin A supplementation in infants and children 6–59 months	0.077	0.394	0.725	0.001	0.002	0.003	1,118,095	Incidence of vitamin A deficiency among children 6–59 months
Pneumonia treatment (children)	0.273	0.502	0.722	0.004	0.007	0.008	462,121	incidence of pneumonia among children under-5
Zinc	0.057	0.089	0.111	0.000	0.001	0.001	1,242,620	Incidence of nutritional deficiencies

## Methods

We model a hypothetical health care system in the AFR-E region with a population of 25 million and an assumed exogenously fixed discretionary budget of $15 million (2015 US) that may be spent on any combination of the 16 interventions listed above at coverage levels of 0%, 50%, 55%, 60%, 65%, 70%, 75%, 80%, 85%, 90%, 95% and 100%. Interventions are assumed to be independent of each other, while coverage levels are mutually exclusive. Note that this budget represents only the discretionary part of total health system spending, which is otherwise assumed to be fixed. $15 million (2015 US) represents on average 15% of total domestic government health expenditure for countries in the region (World Bank Group, 2019). We first present a scenario that allows coverage levels to vary, then extend that scenario to apply differential equity weights to the benefits of coverage, and finally for comparison illustrate a scenario where interventions are either provided at 95% coverage as documented in the WHO-CHOICE data or not at all. In the scenario where differential weights are applied, we assume for the purposes of illustration that patients who are the most difficult to reach are also the poorest, and apply a weight of 4 to the benefits in the last decile of coverage and a weight of 2 to patients in the penultimate decile.^3^ These weights are of course a policy choice, and can readily be amended.

The optimization process works by ranking intervention coverage options by incremental cost effectiveness ratio (ICER), where different levels of the breadth of coverage of an intervention are mutually exclusive, and including them in a stepwise fashion until either the budget is reached or the next most cost effective intervention coverage interval cannot be included without going over budget. Each intervention is covered up to a 5% increment except for the final intervention included, which may be partially included. The steps required for the optimization are detailed below.

Step 1: We first calculate ICERs for each intervention and 5% coverage level alternative using cumulative costs and benefits. As is standard in calculating ICERs, we eliminate any alternative that is less effective and more costly than the next incremental 5% coverage level alternative (to avoid overestimating the cost-effectiveness of the 5% coverage level alternative). We then eliminate any alternative that has a higher ICER than the next incremental 5% coverage level alternative. These are considered “dominated” as more health benefit is achieved with less cost by the next incremental 5% coverage level alternative. When a coverage level alternative is removed, the costs and benefits for the next alternative are calculated incremental to the last alternative that was not removed from the analysis.^4^ We refer to the resulting ICERs as “non-dominated intervention coverage alternatives”.^5^

Under scenario 1, benefits for all interventions are constant or decreasing with coverage and unit costs are non-decreasing with coverage, so the ICERs are generally known to be non-decreasing with coverage. (ICERs calculated for scenario 1 are given in Data Appendix 2, Table 6) In contrast, under scenario 2, both costs and benefits may increase with coverage. As previously discussed, we consider it impractical to allow ‘gaps’ in coverage, whereby certain intermediate coverage groups are denied access. Because benefits at higher levels of coverage are weighted in scenario 2, some incremental increases in coverage for some interventions may be dominated by increases to higher levels of coverage, with the result that some incremental coverage increases of 5% are considered dominated and eliminated and therefore are absorbed into larger incremental increases. For example, it would not be cost-effective to increase management of serious newborn infections from 65% to 70% coverage, nor from 70% to 75%, and so on, until reaching 100% coverage and so only ICERs 0% to 50%, 50% to 55%, 55% to 60%, 60% to 65% and 65–100% for management of serious newborn infections are included in this step of the optimization. (ICERs calculated for scenario 2 are given in Data Appendix 2, Table 7.) Under scenario 3 cost and benefit functions are assumed to be linear as the only known points are a do nothing scenario (with zero cost and zero benefit) and 95% coverage. (ICERs calculated for scenario 3 are given in Data Appendix 2, Table 8.)

Step 2: Rank the non-dominated intervention coverage alternatives by ICER and include these from most to least cost-effective until the budget is reached or the next most cost-effective non-dominated intervention coverage alternative cannot be included without going over the budget. The budget impact for each incremental decision is the per patient cost at the incremental coverage level multiplied by the number of patients the increase would cover. For example, the budget impact of increasing management of serious newborn infections from 55% to 60% would be per patient cost of increasing from 55% to 60% multiplied by 5% of the patient population. The budget impact of increasing from 60% to 100% coverage would be the sum of the budget impact for each 5% marginal increase within that range (as incremental per patient costs may differ for each 5% increment).

Step 3: It is likely that there will be a gap between expenditure on the last included non-dominated intervention coverage alternatives and the budget. We call this gap the budget underspend. The third and final step is to ensure this underspend is spent in a manner that results in the greatest possible gains in overall population health. This step can be broken down into its constituent parts:

• Calculate the budget underspend.
• Eliminate any intervention coverage alternatives for which the additional cumulative expenditure required to include them exceeds the budget underspend.
• Re-calculate ICERs for remaining intervention coverage alternatives (both those that were previously dominated and those that were not).
• Re-rank remaining non-dominated intervention coverage alternatives.
• The algorithm returns to step 1 and is repeated until the remaining budget underspend is less than the expenditure required to scale up any intervention to the next coverage level alternative (dominated or non-dominated).
• The final step is to calculate the ICER of increasing coverage from the current level covered to the next level for each intervention and partially scale up the intervention with the lowest from among these ICERs.

## Results

**Fig. 2:**
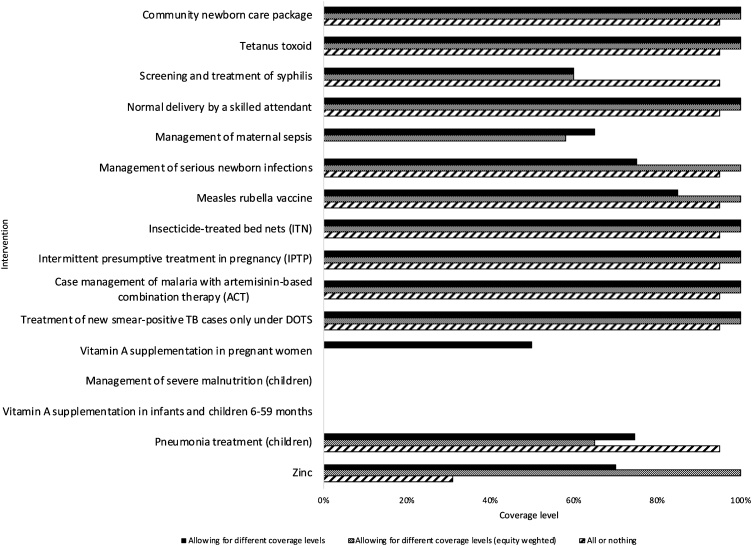
Coverage levels for each intervention.

Fig. 2 shows the results of the optimization for each scenario 1) differential coverage levels are allowed; 2) differential coverage different coverage levels are allowed and differential equity weights are applied to the benefits of coverage; and 3) interventions are either provided at 95% coverage or not at all.

When differential coverage levels are allowed without equity weights (scenario 1) 14 interventions are covered: seven are fully covered at 100% (community newborn care package, tetanus toxoid, normal delivery by a skilled attendant, insecticide treated nets (ITN), intermittent presumptive treatment in pregnancy (IPTP), case management of malaria with artemisinin-based combination therapy (ACT) and treatment of new smear-positive TB cases only under DOTS) and seven at varying lower coverage levels (treatment of syphilis at 60%, management of maternal sepsis at 65%, management of serious newborn infections at 75%, measles rubella vaccine at 85%, vitamin A supplementation in pregnant women at 50%, pneumonia treatment (children) at 75% and zinc at 70%). The last non-dominated intervention coverage alternative included in its entirety is management of maternal sepsis at 65% with an ICER of $1,153 (US 2015). There is a budget underspend of $236,642, and the next most cost-effective non-dominated intervention coverage alternative is pneumonia treatment (children) at 75%, so that is scaled up until the budget is exhausted (i.e., to 74.62%).

When differential equity weights are applied to the benefits of coverage (scenario 2) 13 interventions are included and there is less variation in the levels at which they are covered: ten are fully covered at 100% (the same seven interventions as in scenario one plus management of serious newborn infections, measles rubella vaccine and zinc) and the remaining three are included at 65% (screening and treatment of syphilis), 58% (management of maternal sepsis) and 75% (pneumonia treatment in children). This is because applying differential weights to benefits for the ultimate and penultimate deciles of covered patients has the effect of reducing the ICERs for increasing coverage to these groups, which makes expanding coverage to these groups more appealing than covering other interventions at lower levels. For example, covering vitamin A supplementation in pregnant women at 50% has an ICER of $976. Without equity weights, this is more cost-effective than covering measles rubella vaccine at 85% or any higher level, and indeed under scenario 1 measles rubella vaccine is covered at 85% with an ICER of $1,097. However, when equity weights are applied, expanding measles rubella vaccine beyond 85% is more cost-effective than providing vitamin A supplementation at 50%. Similarly, once equity weights are applied, coverage of management of maternal sepsis and pneumonia treatment (children) are reduced, while coverage of zinc and management of serious newborn infections are expanded. The marginal intervention in this scenario, management of maternal sepsis, has an equity weighted ICER of $900.

In the all or nothing scenario 12 of the 16 interventions are included at 95% apart from zinc as the remaining budget is only enough to include it at 31%. This includes all seven of the interventions covered at 100% in scenario 1. Compared to scenario 1, coverage increases to 95% for screening and treatment of syphilis, management of serious newborn infections, measles rubella vaccine and pneumonia treatment (children). Two interventions that were included in scenario 1 are not included in this scenario: management of maternal sepsis and vitamin A supplementation in pregnant women. No coverage level for either of these interventions was particularly cost-effective in scenario 1 (i.e. not among top intervention coverage alternatives). Moreover, the costs of providing these interventions increase rapidly with coverage level, in particular for vitamin A supplementation.

Table 2 presents the DALYs averted by each intervention given the level of coverage it is included at and the total DALYs averted for each scenario. The total DALYs averted by the included interventions is highest when coverage levels are allowed to vary (i.e., scenario 1: 205,636 DALYs averted) compared to when equity weights are applied (i.e., scenario 2: 205,227) or when interventions decisions are made on the basis of including either at 95% or not at all (i.e., scenario 3: 197,516 DALYs averted). Using equity weights, however, changes the optimal package so that fewer interventions are included overall, but among those more are included at 100%. The optimal package where benefits are equity weighted averts more equity weighted DALYs (273,300) than the optimal package where benefits are not equity weighted (which averts 272,117 equity weighted DALYs), and both avert more equity weighted DALYs than the package where interventions are either included at 95% or not at all (241,771).

**Table 2:** Health effects of intervention at coverage level included at.

	Allowing for different coverage levels				Allowing for different coverage levels (equity weighted)				All or nothing			
Intervention	ICER at coverage level	Coverage level	DALYs averted	Equity weighted DALYs averted	Equity weighted ICER at coverage level	Coverage level	DALYs averted	Equity weighted DALYs averted	ICER at coverage level	Coverage level	DALYs averted	Equity weighted DALYs averted
Community newborn care package	108	100%	595	833	58	100%	595	833	80	95%	565	714
Tetanus toxoid	686	100%	9,925	13,895	224	100%	9,925	13,895	163	95%	9,429	11,910
Screening and treatment of syphilis	776	60%	321	321	776	60%	321	321	954	95%	509	643
Normal delivery by a skilled attendant	674	100%	4,998	6,998	271	100%	4,998	6,998	358	95%	4,748	5,998
Management of maternal sepsis	1,153	65%	82	82	900	58%	70	70				
Management of serious newborn infections	1,072	75%	46	46	684	100%	62	87	555	95%	59	74
Measles rubella vaccine	1,097	85%	4,192	4,373	552	100%	4,661	5,912	380	95%	4,517	5,336
Insecticide-treated bed nets (ITN)	387	100%	10,706	13,903	210	100%	10,706	13,903	269	95%	10,328	12,391
Intermittent presumptive treatment in pregnancy (IPTP)	1,077	100%	141	189	355	100%	141	189	306	95%	135	166
Case management of malaria with artemisinin-based combination therapy (ACT)	44	100%	154,460	205,147	15	100%	154,460	205,147	17	95%	148,346	180,692
Treatment of new smear-positive TB cases only under DOTS	175	100%	15,402	21,563	64	100%	15,402	21,563	70	95%	14,632	18,482
Vitamin A supplementation in pregnant women	976	50%	863	863								
Management of severe malnutrition (children)												
Vitamin A supplementation in infants and children 6–59 months												
Pneumonia treatment (children)	1,164	75%	3,035	3,035	827	65%	2,643	2,643	721	95%	3,863	4,880
Zinc	1,092	70%	870	870	708	100%	1,243	1,740	961	31%	385	486
Total			205,636	272,117			205,227	273,300			197,516	241,771
Maximum ICER	1,164				900				961			

In scenario 1 where coverage levels are allowed to vary (but equity weights are not used) 14 interventions of the 16 are included. The cost-effectiveness ratio of the last included intervention, pneumonia treatment (children) at nearly 75%, is $1,164 (2015 US), and so this represents the marginal productivity of the discretionary budget.

## Discussion

This illustrative example shows how policy makers can be provided with information on where the greatest health gains can be achieved from available coverage options and how this can be traded-off with gains in equity. The first scenario assumes that health gains are valued equally irrespective of characteristics of the beneficiaries. However, policy makers might value health gains of underserved rural/remote populations more highly. One option is for the implications of this to be explored in a deliberative manner when the analysis is being translated into practice, e.g., as was done for the 2017–2022 Malawi Essential Health Package (Ochalek et al., 2018a). As an alternative, Scenario 2 incorporates equity weights explicitly by allowing *w(.)* to vary.

Health benefits packages usually promise a set of interventions for the full population, although these are often only partially available in practice. The promise of full coverage is aligned with the definition of UHC; however, if implicit rationing is taking place (e.g., where the least well off are less able to secure access to care than the most well off) the package is not in reality provided at full coverage. The possible equity gains to be had from directing expenditure toward improving coverage of interventions as opposed to expanding the package can now be quantified, making this trade-off explicit.

Although the method could in principle be used to develop a benefits package from a zero base, this illustrative example addresses a specific, not unusual, decision context where an amount of money is made available to be allocated for discretionary funding of a limited set of possible interventions. This method is therefore appropriate for example when a budget is set aside for allocating resources within a disease-specific budget silo. However, it is important to note that this use of a dedicated budget will not in general be optimal and can lead to health and welfare losses overall. Understanding of this issue was reflected in the interest in sector wide approaches (SWAps) introduced in the 1990s, which provided a mechanism for coordinating funding from disparate sources and allocating resources centrally. However, in practice, appropriate data for all interventions at every possible coverage level is unlikely to exist, and the administrative complexity would in any case be daunting, so the limited optimization we present reflects the usual context in which coverage decisions are made.

Estimates of the marginal productivity of the health care systems in the AFR-E region range from $59 to $5,014 (2015 US) per DALY averted (Ochalek et al., 2018b). Comparisons between these estimates and the $1,164 cost-effectiveness of the last included intervention in our illustrative example (the marginal productivity of the discretionary budget) are difficult for a number of reasons. Whether the marginal productivity of the discretionary budget is higher or lower than a plausible empirical estimate of the marginal productivity of the wider health care system depends upon the representativeness of the interventions that the discretionary budget may be spent on compared to the interventions funded within the wider health care system. If these 16 interventions are more (less) cost-effective than the interventions funded within the wider health care system the marginal productivity of the discretionary budget will be higher (lower) as represented by a lower (higher) cost per DALY averted than that of the wider health care system.

There is no consideration of service quality variations, and in practice we are assuming that all coverage is equally effective. Ineffective coverage (for example, use of vaccines rendered ineffective due to lack of refrigeration) lies outside the definition of UHC and should be considered a type of inefficiency. We further assume under Scenario 1 that for most treatments the incremental health gains secured are equal for all recipients (or exhibit modest reductions as coverage increases). In practice there may be considerable variations in health gains between social groups, for example if the treatment under consideration has different treatment ‘comparators’ in rural and urban areas. Such data is not currently available. However, from a methodological perspective, the possibility of health gains varying systematically according to levels of disadvantage is equivalent to the adoption of equity weights under Scenario 2, so is readily accommodated if relevant data become available.

More generally, as with most such applications, this illustration is replete with uncertainty. In principle the model could be extended by attaching probability distributions to the parameters used, and undertaking stochastic modelling using approaches such as Monte-Carlo simulation. Whilst this would undoubtedly yield valuable further information, it is not clear to what extent it would further assist decision-makers. It is likely that – in practice – most decision-makers would want to focus on a small number of crucial choices relating to treatments for which the cost-effectiveness is at a critical level, the reliability of the data is questionable, there are additional contextual factors to consider, or the budget impact is high. These are legitimate influences on decisions. The role of the analysis is to inform the decision in a transparent, rigorous and systematic way, and not to determine the outcome.

## Conclusion

This paper has offered an operational approach towards the optimal design of the discretionary part of a UHC benefits package. It is based on a theoretical mathematical programme that seeks to reconcile the concepts of depth and breadth coverage, as expressed in the WHO universal health coverage ‘cube’. We note that in practice population coverage is usually less than 100%, even in mature systems of UHC, and that this is likely to arise on the demand side because of user charges and other barriers to access, and on the supply side due to the relatively high costs of providing services for remote or disadvantaged populations.

In order to make the analysis tractable, we have chosen to remove the possibility of imposing user charges, and instead focus on the common supply-side policy problem of the trade-off between breadth and depth of coverage. We use WHO-CHOICE data to demonstrate how an optimal solution can be secured. With a notional $15 million budget available, it indicates that 205,636 DALYs could be averted compared to the 197,516 DALYs averted if decision-makers insist that (almost) full coverage should be secured for all interventions in a necessarily narrower package. We show the opportunity cost (in the form of lost DALYs averted) of choosing to expand levels of population coverage (breadth of coverage) at the expense of the range of treatments offered (depth of coverage), and how – if desired – equity weights can be introduced into the optimization.

The paper adapts the principles of conventional cost-effectiveness analysis to situations in which estimates of both costs and benefits may vary depending on the levels of population coverage achieved. Therefore the analysis suffers from many of the acknowledged limitations of many CEA studies. In common with usual CEA practice, the interventions are considered to be independent of each other, and we do not allow for the possibility of economies of scope. This issue is recognized as being an important limitation of conventional CEA, and may be one of the reasons why unit costs are lower in urban centres, particularly for hospital-based services. Methodologies to accommodate economies of scope within CEA are beginning to emerge, and should in principle be incorporated into benefit package design (Hauck et al., 2019). However, they are at an early stage of development. Finally, we consider just one period, and do not examine the multiperiod implications of coverage decisions (apart from the future health benefits attained). It may well be the case that future research into some of these limitations will be valuable, but for the current paper we choose to remain consistent with conventional CEA practice, apart from allowing costs and benefits to vary according to levels of coverage.

A more immediate limitation in our view is the general lack of persuasive cost-effectiveness data relating to coverage levels and how they correspond to levels of wealth. We opportunistically took advantage of WHO-CHOICE calculations to demonstrate the methods needed to assess optimal coverage levels. The production of these data is a big step forward. However, even the WHO data are broad estimates, the methodology on which the cost functions are based is not transparent, and they are not country-specific. Moreover, in practical decision-making situations, such data will be almost entirely lacking for many interventions and country settings. Implicitly, the high costs of reaching disadvantaged populations are likely to be an important reason for the low levels of coverage found in many countries, and yet we are not aware of many datasets that quantify unit cost variations, which would allow decision-makers to make informed decisions about the trade-off between breadth and depth of coverage. We see the provision of data on variations in unit costs and health benefits at the country level as a priority for future empirical research in this area. Whilst in many respects daunting, the provision of such data may be a feasible and relatively low-cost extension of conventional CEA studies, and we recommend that sponsors of such research give serious consideration to funding disaggregated data that would inform the pursuit of equity in health care.

We hope that – by illustrating how realistic coverage decisions could in principle be made – we can stimulate more research into the data and analytic methods necessary to inform such decisions. The policy choice of whether or not to tolerate some element of ‘unmet need’ in a system of UHC is an agonizing one, with profound consequences for those who are denied treatment. However, we have shown on the other hand that insistence on pursuing high population coverage levels without regard to the implications for the size of the benefits package also has serious opportunity costs, in the form of the health benefits from treatments foregone because they could not be included in the benefits package. We are under no illusions about the challenges raised by seeking to apply analytic methods in this area. But the methods described do offer the opportunity to help decision-makers become more aware of the structure of the coverage problem they confront, the data that are needed to inform analytic models, and the population health consequences of their coverage choices.

## Funding

This work was supported by UK Research and Innovation as part of the Global Challenges Research Fund, grant number MR/P028004/1, 2017.

## CRediT authorship contribution statement

**Jessica Ochalek:** Methodology, Data curation, Formal analysis, Writing - original draft. **Gerald Manthalu:** Writing - review & editing. **Peter C. Smith:** Conceptualization, Methodology, Writing - original draft, Supervision.

## Footnotes

1 Botswana, Burundi, Central African Republic, Congo, Côte d'Ivoire, Democratic Republic of the Congo, Eritrea, Ethiopia, Kenya, Lesotho, Malawi, Mozambique, Namibia, Rwanda, South Africa, Swaziland, Uganda, United Republic of Tanzania, Zambia and Zimbabwe.

2 Incidence of disease and population size: Global Burden of Disease (http://ghdx.healthdata.org/gbd-results-tool) and percentage of women 15–49 currently pregnant: Demographic and Health Surveys Stat Compiler tool (https://www.statcompiler.com/en/) both accessed 01/02/2019.

3 Another method of incorporating equity is through the use of indirect equity weights or aggregate distributional cost-effectiveness analysis. This method requires an estimate of the health opportunity costs of changes in health expenditure and its distribution across relevant population subgroups. Where the budget is known, but opportunity costs are undefined, a direct equity weighting approach is more feasible.

4 For details on CEA methods for calculating ICERs see Drummond et al., 2015 and Sculpher et al., 2017.

5 Dominated alternatives include both strongly and extendedly (or weakly) dominated alternatives.
