# Supplementary material for: Squaring the cube: Towards an operational model of optimal universal health coverage
Source: J Health Econ. 2020 Mar;70:102282. doi: 10.1016/j.jhealeco.2019.102282 (PMC7188249; doi:10.1016/j.jhealeco.2019.102282)
Supplement: Supplementary file 1 [file mmc1.docx]

**Appendix 1: A theoretical framework for UHC optimization**

The WHO ‘cube’ illustrates diagrammatically the three broad dimensions along which policy choices must be made as countries progress towards universal health coverage:

1. Services: which services should be covered?
2. Population: which population groups should be covered?
3. Direct costs: what proportion of the costs of access are covered?

The purpose of the cube is to highlight the trade-offs that policymakers must make in seeking to allocate a fixed budget of pooled health service funds. To support this process, analytic attention has focused mainly on (1), in the form of the design of the health benefits package. Far less attention has been paid to (2) and (3). Yet in practice, policy makers are often faced with hard choices relating to (2) and (3). For example, to what extent should the goal of universal (100%) coverage for a specific service be pursued when the costs of reaching vulnerable or remote populations greatly exceed the costs of reaching the general population, and the achievement of such 100% coverage may preclude inclusion of other services in the benefits package? In short, there may be a fundamental trade-off between equity and efficiency in the design and implementation of the benefits package.

This paper offers an analytic framework for examining that trade-off. It seeks out the socially optimal set of services, levels of coverage and user charges for a fixed government budget *X*, extending the framework suggested by Smith (2005, 2013). The population is heterogeneous in wealth *y*, distributed according to a density function $\gamma(y)$. It is assumed that there is a set of *N* efficient health treatments *i*, each of which addresses a discrete health problem, and creates a health benefit for the individual of *b_i_*, which may be expressed in the form of quality-adjusted life years. The costs of supplying the treatment *x_i_(y)* are assumed to be non-increasing in *y*, reflecting the additional costs often associated with delivering effective treatments to disadvantaged groups $x_{i}^{'}(y)\leq0$. We also allow for the potential for variable epidemiology of each disease by allowing the incidence – the probability of needing treatment $\pi_{i}(y)$ - to vary with wealth. Throughout we assume a single time period (although of course the benefits of treatment may extend well beyond that period).

On the demand side we adopt a simple concept of individual utility that is linear in health (as is implicit in traditional cost-effectiveness analysis) and is separable in health and wealth, such that *u(h,y) = h + v(y)*, where *h* is the individual’s level of health and *y* her wealth. We assume *v(.)* has the usual properties $v^{'}(.)\geq0$ and $v^{''}(.)\leq0$ When a health shock arises, the individual is assumed to accept treatment if the value of the health benefits of treatment *b_i_* exceeds the impact of its price to the individual *p_i_*, that is $b_{i}\geq v\left( y \right)-v\left( y-p_{i} \right)$. Conversely, treatment is foregone if the health benefits do not compensate for the impact of the price of access, that is $b_{i}\leq v\left( y \right)-v\left( y-p_{i} \right)$. In this formulation we assume an equal price for all individuals.

For each treatment there is a critical level of wealth $\psi(p_{i})$ at which there is indifference between treatment and no treatment with price *p_i_*, so that:

$$b_{i}+v\left( \psi\left( p_{i} \right)-p_{i} \right)=v\left( \psi\left( p_{i} \right) \right)$$

Demand for treatment with price *p_i_* is then:

$$\theta_{i}\left( p_{i} \right)=\int_{\psi\left( p_{i} \right)}^{\infty} \pi_{i}\left( y \right)\gamma\left( y \right)dy$$

$$\theta_{i}^{'}\left( p_{i} \right)=-\pi_{i}\left( \psi\left( p_{i} \right) \right).\gamma\left( \psi\left( p_{i} \right) \right).\psi^{'}\left( p_{i} \right)\leq0$$

In order to reflect equity concerns, a policy weight *w(y)* is attached to a person at each level of wealth *y*, with the assumption that this is skewed in favour of disadvantaged populations, $w_{i}^{'}(y)\leq0$. We then assume that the social welfare function comprises the (weighted) sum of individual utility losses associated with a set of payments *p_i_*:

$$SWL=\sum_{i} \left\{ \int_{0}^{\psi\left( p_{i} \right)} b_{i}w(y)\pi_{i}\left( y \right)\gamma\left( y \right)dy+\int_{\psi\left( p_{i} \right)}^{\infty} \left[ v\left( y \right)-v(y-p_{i}) \right]w(y)\pi_{i}\left( y \right)\gamma\left( y \right)dy \right\}$$

The first term is the expected health loss of those who do not secure access to care, and the second reflects the financial loss of those who do use care.

The costs of providing treatment (net of any user fee income) are constrained by the availability of public funds *X*:

$$\sum_{i} \int_{\psi\left( p_{i} \right)}^{\infty} \left[ x_{i}(y)-p_{i}) \right]\pi_{i}\left( y \right)\gamma\left( y \right)dy\leq X$$

There is in addition a constraint on the value each price *p_i_* can take, which is no greater than the market price *M_i_* so that $0\leq p_{i}\leq M_{i}$.

Consider first the case in which no user fees can be charged, the prime focus of the case study presented in this paper. Then the policy problem is the trade-off between maximizing coverage and maximizing aggregate health gain, according to the chosen social welfare function. In this case, the absence of user fees implies formulating the problem as:

$$\mathrm{Minimize}\sum_{i} \int_{0}^{z_{i}} b_{i}w(y)\pi_{i}\left( y \right)\gamma\left( y \right)dy subject to\sum_{i} \int_{z_{i}}^{\infty} x_{i}(y)\pi_{i}\left( y \right)\gamma\left( y \right)dy\leq X$$

The decision variables $z_{i}$ indicate the wealth level below which treatment cannot be offered for treatment *i* (because the benefits of reaching the disadvantaged populations are outweighed by the costs to the rest of the health system). For interior solutions this leads to first order conditions for each treatment *i*:

$$b_{i}w\left( z_{i}^{*} \right)\pi_{i}\left( z_{i}^{*} \right)\gamma\left( z_{i}^{*} \right)=\lambda x_{i}\left( z_{i}^{*} \right)\pi_{i}\left( z_{i}^{*} \right)\gamma\left( z_{i}^{*} \right)$$

where $\lambda$ is the Lagrange multiplier. This can be rewritten $b_{i}w\left( z_{i}^{*} \right)/x_{i}\left( z_{i}^{*} \right)=\lambda$, indicating that the critical level of wealth (and therefore coverage) for each treatment is determined by the ratio of (equity weighted) benefits to production costs. This is the standard cost-effectiveness rule, except that costs are allowed to vary with wealth, and therefore it may be the case that some disadvantaged groups are not covered by the entire health benefits package enjoyed by wealthier populations.

If the entire population is covered $z_{i}^{*}=0$, then the condition becomes $b_{i}w\left( 0 \right)/x_{i}\left( 0 \right)\geq\lambda$. When none are covered by publicly funded services, only those who are prepared to pay the market rate *M_i_* will secure access, then $b_{i}w\left( z_{i}^{*} \right)/x_{i}\left( z_{i}^{*} \right)\leq\lambda$.

For completeness we present the case when user charges are feasible. The fundamental decision variables for the policy maker are then the out of pocket payment levels pi for each treatment i. The associated Lagrangean is:

$$\mathcal{L}\left( \boldsymbol{p},\lambda, \boldsymbol{\mu},\boldsymbol{\nu} \right)=\sum_{i} \left\{ \int_{0}^{\psi\left( p_{i} \right)} b_{i}w\left( y \right)\pi_{i}\left( y \right)\gamma\left( y \right)dy+\int_{\psi\left( p_{i} \right)}^{\infty} \left[ v\left( y \right)-v\left( y-p_{i} \right) \right]w\left( y \right)\pi_{i}\left( y \right)\gamma\left( y \right)dy \right\}$$

$$+\lambda\left\{ \sum_{i} \int_{\psi\left( p_{i} \right)}^{\infty} \left[ x_{i}(y)-p_{i}) \right]\pi_{i}\left( y \right)\gamma\left( y \right)dy-X \right\}+ \sum_{i} {\mu_{i}(p}_{i}-M_{i})-\sum_{i} \nu_{i}p_{i}$$

Differentiation with respect to payments *p_i_* yields:

$$\frac{\partial L}{\partial p_{i}}=b_{i}.w\left( \psi\left( p_{i} \right) \right).\pi_{i}\left( \psi\left( p_{i} \right) \right).\gamma\left( \psi\left( p_{i} \right) \right).\psi^{'}\left( p_{i} \right)$$

$$-[v\left( \psi\left( p_{i} \right) \right)-v\left( \psi\left( p_{i} \right)-p_{i} \right)].w\left( \psi\left( p_{i} \right) \right).\pi_{i}\left( \psi\left( p_{i} \right) \right).\gamma\left( \psi\left( p_{i} \right) \right).\psi^{'}\left( p_{i} \right)$$

$$+\int_{\psi\left( p_{i} \right)}^{\infty} v^{'}\left( y-p_{i} \right)w\left( y \right)\pi_{i}\left( y \right)\gamma\left( y \right)dy$$

$$-\lambda\int_{\psi\left( p_{i} \right)}^{\infty} \pi_{i}\left( y \right)\gamma\left( y \right)dy$$

$$-\lambda\left[ x_{i}(\psi\left( p_{i} \right))-p_{i}) \right].\pi_{i}\left( \psi\left( p_{i} \right) \right).\gamma\left( \psi\left( p_{i} \right) \right).\psi^{'}\left( p_{i} \right)$$

$$+\mu_{i}-\nu_{i}$$

The terms in this expression reflect the consequences of a marginal increase in copayment, in turn as follows:

1. The loss of health benefits to marginal citizens foregoing treatment;
2. The gain in wealth-related utility to marginal citizens foregoing treatment;
3. The loss of wealth-related utility amongst all users of the treatment;
4. The social gain of reduced public expenditure on remaining users;
5. The reduction in social expenditure on the marginal citizens foregoing treatment
6. The impact of the practical constraints on *p_i_*.

This formulation leads to three classes of solution for the values of each *p_i_*: treatments for which the payment is zero (access can be secured free of charge); treatments for which there is no public subsidy (so access can be secured only by payment of the full market price *M_i_*); and intermediate solutions for which some (but not full) public subsidy is optimal. We consider the nature of each solution in turn and examine only first order conditions. The functions $\theta_{i}(p_{i})$ are not in general concave, so these results only describe necessary characteristics of an optimal solution.

Note that it is assumed that – with no user charges and no restrictions on access – demand would reflect total population need:

$$\theta_{i}\left( 0 \right)=\int_{0}^{\infty} \pi_{i}\left( y \right)\gamma\left( y \right)dy$$

and the level of coverage given price *p_i_* is $\theta_{i}\left( p_{i} \right)/\theta_{i}\left( 0 \right)$. Therefore, implicitly, by choosing user charges, the policy maker is also choosing the level of coverage for each treatment.

When optimal price is zero, *p_i_^*^* = 0; *μ_i_* = 0; *ν_i_* ≥ 0 and the optimization simplifies to:

$$\frac{\partial\mathcal{L}}{\partial p_{i}}=-w\left( 0 \right).b_{i}.\theta_{i}^{'}\left( 0 \right)+\lambda x_{i}\left( 0 \right).\theta_{i}^{'}\left( 0 \right)-\lambda\theta_{i}\left( 0 \right)+\int_{0}^{\infty} v^{'}\left( y \right)w\left( y \right)\pi_{i}\left( y \right)\gamma\left( y \right)dy-\nu_{i}=0$$

which can be rearranged to yield:

$$-w\left( 0 \right)b_{i}\theta_{i}^{'}\left( 0 \right)+\int_{0}^{\infty} v^{'}\left( y \right)w\left( y \right)\pi_{i}\left( y \right)\gamma\left( y \right)dy\geq-\lambda x_{i}\left( 0 \right)\theta_{i}^{'}\left( 0 \right)+\lambda\theta_{i}\left( 0 \right)$$

The left hand side of this expression shows the losses caused by a marginal increase in user fee from the zero optimum. These comprise (a) the (weighted) loss of health benefits to those denied treatment by the increase and (b) the (weighted) loss of financial utility suffered by all remaining users caused by the fee increase. The right hand side shows the equivalent societal gains, arising from (c) the reduction in demand (and therefore public expenditure) caused by the marginal increase in price and (d) the increased fee income deriving from the increased user charge. In short, the inequality shows that the benefits of any reduction in public expenditure secured by a user fee are outweighed by the diminished utility levels expressed in terms of reduced health and increased private payment.

When subsidy is zero, *p_i_^*^* = *M_i_*, *μ_i_* ≥ 0; *ν_i_* = 0 and the optimization simplifies to:

$$\frac{\partial\mathcal{L}}{\partial p_{i}}=-b_{i}.w\left( \psi\left( M_{i} \right) \right).\theta_{i}^{'}\left( M_{i} \right)+[v\left( \psi\left( M \right) \right)-v\left( \psi\left( M_{i} \right)-M_{i} \right)].w\left( \psi\left( M_{i} \right) \right).\theta_{i}^{'}\left( M_{i} \right)$$

$$+\int_{\psi\left( M_{i} \right)}^{\infty} v^{'}\left( y-p_{i} \right)w\left( y \right)\pi_{i}\left( y \right)\gamma\left( y \right)dy-\lambda\int_{\psi\left( M_{i} \right)}^{\infty} \pi_{i}\left( y \right)\gamma\left( y \right)dy+\mu_{i}$$

We assume production costs equal the private equilibrium market fees at the critical wealth level (so that $x_{i}\left( \psi\left( M_{i} \right) \right)=M_{i}$).

Rearranging to place the gains from a price reduction on the left hand side and the losses on the right hand side, we get:

$$-b_{i}.w\left( \psi\left( M_{i} \right) \right).\theta_{i}^{'}\left( M_{i} \right)+\int_{\psi\left( M_{i} \right)}^{\infty} v^{'}\left( y-M_{i} \right)w\left( y \right)\pi_{i}\left( y \right)\gamma\left( y \right)dy\leq$$

$$-[v\left( \psi\left( M \right) \right)-v\left( \psi\left( M_{i} \right)-M_{i} \right)].w\left( \psi\left( M_{i} \right) \right).\theta_{i}^{'}\left( M_{i} \right)+\lambda\int_{\psi\left( M_{i} \right)}^{\infty} \pi_{i}\left( y \right)\gamma\left( y \right)dy$$

The left hand side of this expression shows the gains arising from a marginal decrease in user fee from the optimum of 100% fee. These comprise (a) the (weighted) gain in health benefits to those able to secure treatment because of the decrease and (b) the (weighted) gain in financial utility enjoyed by all users caused by the fee reduction. The right hand side shows the equivalent societal losses, arising from (c) the increase in demand (and therefore public expenditure) caused by the marginal reduction in price and (d) the subsidy for all users arising from the reduced user charge. The inequality indicates that the gains in utility derived from a reduction in user fee (the left hand side) are outweighed by the societal costs of the reduction.

When an intermediate solution is optimal, *M_i_* > *p_i_^*^* > 0 and *μ_i_* = 0; *ν_i_* = 0. Then:

$$0=\int_{\psi\left( p_{i} \right)}^{\infty} v^{'}\left( y-p_{i} \right)w\left( y \right)\pi_{i}\left( y \right)\gamma\left( y \right)dy-\lambda\int_{\psi\left( p_{i} \right)}^{\infty} \pi_{i}\left( y \right)\gamma\left( y \right)dy$$

$$-\lambda\left[ x_{i}(\psi\left( p_{i} \right))-p_{i}) \right].\pi_{i}\left( \psi\left( p_{i} \right) \right).\gamma\left( \psi\left( p_{i} \right) \right).\psi^{'}\left( p_{i} \right)$$

The expression reflects, in turn, the marginal impact of an increase in user fee on: (a) the loss of wealth-related utility amongst treatment users, due to the increased fees; (b) the associated reduction in public expenditure on all remaining users; and (c) the reduced public expenditure associated with patients deterred from treatment at the margin.

Rearranging indicates that, at this optimum, the individual loss of utility amongst service users brought about by a fee increase must equal the societal gains from increased fee income and the reduced utilization (and therefore public expenditure) caused by the increased fee.

$$\int_{\psi\left( p_{i} \right)}^{\infty} v^{'}\left( y-p_{i} \right)w\left( y \right)\pi_{i}\left( y \right)\gamma\left( y \right)dy=\lambda\theta_{i}(p_{i})-\lambda\left[ x_{i}(\psi\left( p_{i} \right))-p_{i}) \right].\theta_{i}^{'}\left( p_{i} \right)$$

Note that, amongst those patients deterred at the margin by the fee increase, the loss of health-related utility is balanced by the gain in wealth-related utility, so neither appears in this equation.

**Appendix 2: Data, methods and results**

Data Appendix Table 1 reports the costs and benefits by 50%, 80% and 95% coverage from WHO-CHOICE and the annual incidence for the a population of 25 million as reported within the text.

| Data Appendix Table 1. WHO-CHOICE costs and benefits by coverage level and patient population | | | | | | | | | | | | | |  | | |
| --- | --- | --- | --- | --- | --- | --- | --- | --- | --- | --- | --- | --- | --- | --- | --- | --- |
|  | Cost per year (I$, millions) per capita | | | | DALYs averted per year per capita | | | | | | Incident population (2015) | | | |  |  |
| Intervention | 50% | 80% | | 95% | | 50% | | 80% | | 95% | | Number | Definition | | |  |
| Community newborn care package | 0.089 | 0.145 | 0.179 | | 0.011 | | 0.017 | | 0.020 | | 27,662 | | Population 0-27 days of age | |  |  |
| Tetanus toxoid | 0.058 | 0.118 | 0.194 | | 0.005 | | 0.007 | | 0.009 | | 1,067,218 | | Population <1 year | |  |  |
| Screening and treatment of syphilis | 0.034 | 0.070 | 0.119 | | 0.000 | | 0.001 | | 0.001 | | 595,029 | | Percentage of women 15-49 currently pregnant * population female 15-49 | |  |  |
| Normal delivery by a skilled attendant | 0.157 | 0.259 | 0.334 | | 0.004 | | 0.007 | | 0.008 | | 595,029 | | Percentage of women 15-49 currently pregnant * population female 15-49 | |  |  |
| Management of maternal sepsis | 0.088 | 0.154 | 0.219 | | 0.001 | | 0.001 | | 0.002 | | 70,148 | | Incidence of maternal sepsis and other maternal infections | |  |  |
| Management of serious newborn infections | 0.150 | 0.268 | 0.403 | | 0.003 | | 0.004 | | 0.005 | | 11,264 | | Incidence of neonatal sepsis and other neonatal infections | |  |  |
| Measles rubella vaccine | 0.100 | 0.162 | 0.224 | | 0.001 | | 0.003 | | 0.003 | | 1,067,218 | | Population <1 year | |  |  |
| Insecticide-treated bed nets (ITN) | 0.474 | 0.629 | 0.710 | | 0.010 | | 0.015 | | 0.017 | | 595,029 | | Percentage of women 15-49 currently pregnant * population female 15-49 | |  |  |
| Intermittent presumptive treatment in pregnancy (IPTP) | 0.054 | 0.057 | 0.060 | | 0.000 | | 0.000 | | 0.000 | | 595,029 | | Percentage of women 15-49 currently pregnant * population female 15-49 | |  |  |
| Case management of malaria with artemisinin-based combination therapy (ACT) | 0.192 | 0.203 | 0.211 | | 0.009 | | 0.015 | | 0.017 | | 8,375,236 | | Incidence of malaria | |  |  |
| Treatment of new smear-positive TB cases only under DOTS | 0.428 | 0.768 | 1.069 | | 0.068 | | 0.109 | | 0.130 | | 112,918 | | Incidence of TB | |  |  |
| Vitamin A supplementation in pregnant women | 0.077 | 0.394 | 0.725 | | 0.001 | | 0.002 | | 0.003 | | 595,029 | | Percentage of women 15-49 currently pregnant * population female 15-49 | |  |  |
| Management of severe malnutrition (children) | 5.039 | 8.085 | 9.651 | | 0.000 | | 0.000 | | 0.000 | | 122,555 | | Incidence of protein-energy malnutrition among children under-5 | |  |  |
| Vitamin A supplementation in infants and children 6-59 months | 0.077 | 0.394 | 0.725 | | 0.001 | | 0.002 | | 0.003 | | 1,118,095 | | Incidence of vitamin A deficiency among children 6-59 months | |  |  |
| Pneumonia treatment (children) | 0.273 | 0.502 | 0.722 | | 0.004 | | 0.007 | | 0.008 | | 462,121 | | incidence of pneumonia among children under-5 | |  |  |
| ORS | 0.687 | 1.220 | 1.669 | | 0.006 | | 0.010 | | 0.012 | |  | |  | |  |  |
| Zinc | 0.057 | 0.089 | 0.111 | | 0.000 | | 0.001 | | 0.001 | | 1,242,620 | | Incidence of nutritional deficiencies | |  |  |

Appendix 2 Figure 1 plots the cost and benefits for community newborn care package based on the WHO-CHOICE data presented in Data Appendix Table 1. The cost function exhibits non-linearity, and so we apply a quadratic relationship. The benefit function is linear. From these, we interpolate the costs and benefits at 55%, 60%, 65%, 70%, 75%, 85%, 90% and 100% coverage.

**Appendix 2 Figure 1. Cost and benefit functions of community newborn care package**

Appendix 2 Table 2 reports the cost and benefit functions where c is the proportion of the patient population covered for all of the interventions used in our illustration.

| Data Appendix Table 2. Costs and benefit functions | |  |  |  |
| --- | --- | --- | --- | --- |
| Intervention | Cost function | **Type** | **Benefit function** | **Type** |
|  |  |  |  |  |
| Community newborn care package | 0.082c^2^ + 0.08c + 0.0288 | quadratic | 0.0215c | linear |
| Tetanus toxoid | 0.6688c^2^ - 0.6685c + 0.225 | quadratic | 0.0093c + 4E-17 | linear |
| Screening and treatment of syphilis | 0.4563c^2^ - 0.4743c + 0.1575 | quadratic | 0.0009c - 1E-18 | linear |
| Normal delivery by a skilled attendant | 0.3653c^2^ - 0.1365c + 0.134 | quadratic | 0.0084c | linear |
| Management of maternal sepsis | 0.4787c^2^ - 0.4046c + 0.1709 | quadratic | 0.0018c + 6E-18 | linear |
| Management of serious newborn infections | 1.1331c^2^ - 1.0814c + 0.4078 | quadratic | 0.0055c - 2E-17 | linear |
| Measles rubella vaccine | 0.4595c^2^ - 0.391c + 0.181 | quadratic | -0.0023c^2^ + 0.0073c - 0.0018 | quadratic |
| Insecticide-treated bed nets (ITN) | 0.5152c + 0.2164 | linear | -0.0073c^2^ + 0.0273c - 0.0022 | quadratic |
| Intermittent presumptive treatment in pregnancy (IPTP) | 0.0196c^2^ - 0.0166c + 0.0577 | quadratic | -5E-05c^2^ + 0.0003c - 2E-05 | quadratic |
| Case management of malaria with artemisinin-based combination therapy (ACT) | 0.0445c^2^ - 0.0223c + 0.1923 | quadratic | -0.0053c^2^ + 0.0252c - 0.0022 | quadratic |
| Treatment of new smear-positive TB cases only under DOTS | 1.9541c^2^ - 1.409c + 0.6442 | quadratic | 0.1364c + 4E-16 | linear |
| Vitamin A supplementation in pregnant women | 2.5418c^2^ - 2.245c + 0.5637 | quadratic | 0.0029c | linear |
| Management of severe malnutrition (children) | 0.6319c^2^ + 9.3321c + 0.2148 | quadratic | 0.0002c | linear |
| Vitamin A supplementation in infants and children 6-59 months | 2.5418c^2^ - 2.245c + 0.5637 | quadratic | 0.0029c | linear |
| Pneumonia treatment (children) | 1.5526c^2^ - 1.2548c + 0.5126 | quadratic | 0.0088c | linear |
| Zinc | 0.0837c^2^ - 0.0027c + 0.0375 | quadratic | 0.001c + 1E-18 | linear |

All of the cost functions exhibit non-linearity to which we apply a quadratic relationship. The exception is ITN distribution to pregnant women, which has a linear cost function. For all other interventions a marginal increase in cost for a marginal increase in coverage is higher for each 5% increment covered, as estimated by WHO. This negative association between costs and coverage reflects the increased cost of reaching disadvantaged populations. Most benefit functions are found to be linear, as expected given harder to reach populations are unlikely to benefit any more or less from treatment than relatively easier to reach populations.^[[1]](#footnote-1)^ However, the WHO report diminishing marginal returns to benefits from increased coverage of measles rubella vaccine; ITN distribution to pregnant women; IPT to pregnant women; and malaria treatment. This may be due to herd immunity effects in the case of the vaccine and the nature of the way malaria is spread in the case of the others.

These approximated relationships are differentiated with respect to coverage to obtain the marginal costs (2000 Int$) and marginal benefits at 50%, 55%, 60%, 65%, 70%, 75%, 80%, 85%, 90%, 95% and 100% coverage. The per patient benefits (per 1,000 patients) are reported in Appendix 2 Table 3.

| Appendix 2 Table 3. Per patient benefit (per 1,000 patients) (i.e. marginal increase in benefit per patient) | | | | | | | | | | | | |
| --- | --- | --- | --- | --- | --- | --- | --- | --- | --- | --- | --- | --- |
| Intervention | 50% | 55% | 60% | 65% | 70% | 75% | 80% | 85% | 90% | 95% | 100% |  |
| Community newborn care package | 21.50 | 21.50 | 21.50 | 21.50 | 21.50 | 21.50 | 21.50 | 21.50 | 21.50 | 21.50 | 21.50 |  |
| Tetanus toxoid | 9.30 | 9.30 | 9.30 | 9.30 | 9.30 | 9.30 | 9.30 | 9.30 | 9.30 | 9.30 | 9.30 |  |
| Screening and treatment of syphilis | 0.90 | 0.90 | 0.90 | 0.90 | 0.90 | 0.90 | 0.90 | 0.90 | 0.90 | 0.90 | 0.90 |  |
| Normal delivery by a skilled attendant | 8.40 | 8.40 | 8.40 | 8.40 | 8.40 | 8.40 | 8.40 | 8.40 | 8.40 | 8.40 | 8.40 |  |
| Management of maternal sepsis | 1.80 | 1.80 | 1.80 | 1.80 | 1.80 | 1.80 | 1.80 | 1.80 | 1.80 | 1.80 | 1.80 |  |
| Management of serious newborn infections | 5.50 | 5.50 | 5.50 | 5.50 | 5.50 | 5.50 | 5.50 | 5.50 | 5.50 | 5.50 | 5.50 |  |
| Measles rubella vaccine | 5.00 | 4.77 | 4.54 | 4.31 | 4.08 | 3.85 | 3.62 | 3.39 | 3.16 | 2.93 | 2.70 |  |
| Insecticide-treated bed nets (ITN) | 20.00 | 19.27 | 18.54 | 17.81 | 17.08 | 16.35 | 15.62 | 14.89 | 14.16 | 13.43 | 12.70 |  |
| Intermittent presumptive treatment in pregnancy (IPTP) | 0.25 | 0.25 | 0.24 | 0.24 | 0.23 | 0.23 | 0.22 | 0.22 | 0.21 | 0.21 | 0.20 |  |
| Case management of malaria with artemisinin-based combination therapy (ACT) | 19.90 | 19.37 | 18.84 | 18.31 | 17.78 | 17.25 | 16.72 | 16.19 | 15.66 | 15.13 | 14.60 |  |
| Treatment of new smear-positive TB cases only under DOTS | 136.40 | 136.40 | 136.40 | 136.40 | 136.40 | 136.40 | 136.40 | 136.40 | 136.40 | 136.40 | 136.40 |  |
| Vitamin A supplementation in pregnant women | 2.90 | 2.90 | 2.90 | 2.90 | 2.90 | 2.90 | 2.90 | 2.90 | 2.90 | 2.90 | 2.90 |  |
| Management of severe malnutrition (children) | 0.20 | 0.20 | 0.20 | 0.20 | 0.20 | 0.20 | 0.20 | 0.20 | 0.20 | 0.20 | 0.20 |  |
| Vitamin A supplementation in infants and children 6-59 months | 2.90 | 2.90 | 2.90 | 2.90 | 2.90 | 2.90 | 2.90 | 2.90 | 2.90 | 2.90 | 2.90 |  |
| Pneumonia treatment (children) | 8.80 | 8.80 | 8.80 | 8.80 | 8.80 | 8.80 | 8.80 | 8.80 | 8.80 | 8.80 | 8.80 |  |
| Zinc | 1.00 | 1.00 | 1.00 | 1.00 | 1.00 | 1.00 | 1.00 | 1.00 | 1.00 | 1.00 | 1.00 |  |

Appendix 2 Table 4 reports the per patient benefits (per 1,000 patients) after equity weights are applied. A weight of 2 is applied to patients in the penultimate decile and a weight of 4 is applied to the benefits in the last decile of coverage.

| Appendix 2 Table 4. Equity weighted per patient benefit (per 1,000 patients) (i.e. marginal increase in benefit per patient) | | | | | | | | | | | |
| --- | --- | --- | --- | --- | --- | --- | --- | --- | --- | --- | --- |
| Intervention | 50% | 55% | 60% | 65% | 70% | 75% | 80% | 85% | 90% | 95% | 100% |
| Community newborn care package | 21.50 | 21.50 | 21.50 | 21.50 | 21.50 | 21.50 | 21.50 | 43.00 | 43.00 | 86.00 | 86.00 |
| Tetanus toxoid | 9.30 | 9.30 | 9.30 | 9.30 | 9.30 | 9.30 | 9.30 | 18.60 | 18.60 | 37.20 | 37.20 |
| Screening and treatment of syphilis | 0.90 | 0.90 | 0.90 | 0.90 | 0.90 | 0.90 | 0.90 | 1.80 | 1.80 | 3.60 | 3.60 |
| Normal delivery by a skilled attendant | 8.40 | 8.40 | 8.40 | 8.40 | 8.40 | 8.40 | 8.40 | 16.80 | 16.80 | 33.60 | 33.60 |
| Management of maternal sepsis | 1.80 | 1.80 | 1.80 | 1.80 | 1.80 | 1.80 | 1.80 | 3.60 | 3.60 | 7.20 | 7.20 |
| Management of serious newborn infections | 5.50 | 5.50 | 5.50 | 5.50 | 5.50 | 5.50 | 5.50 | 11.00 | 11.00 | 22.00 | 22.00 |
| Measles rubella vaccine | 5.00 | 4.77 | 4.54 | 4.31 | 4.08 | 3.85 | 3.62 | 6.78 | 6.32 | 11.72 | 10.80 |
| Insecticide-treated bed nets (ITN) | 20.00 | 19.27 | 18.54 | 17.81 | 17.08 | 16.35 | 15.62 | 29.78 | 28.32 | 53.72 | 50.80 |
| Intermittent presumptive treatment in pregnancy (IPTP) | 0.25 | 0.25 | 0.24 | 0.24 | 0.23 | 0.23 | 0.22 | 0.43 | 0.42 | 0.82 | 0.80 |
| Case management of malaria with artemisinin-based combination therapy (ACT) | 19.90 | 19.37 | 18.84 | 18.31 | 17.78 | 17.25 | 16.72 | 32.38 | 31.32 | 60.52 | 58.40 |
| Treatment of new smear-positive TB cases only under DOTS | 136.40 | 136.40 | 136.40 | 136.40 | 136.40 | 136.40 | 136.40 | 272.80 | 272.80 | 545.60 | 545.60 |
| Vitamin A supplementation in pregnant women | 2.90 | 2.90 | 2.90 | 2.90 | 2.90 | 2.90 | 2.90 | 5.80 | 5.80 | 11.60 | 11.60 |
| Management of severe malnutrition (children) | 0.20 | 0.20 | 0.20 | 0.20 | 0.20 | 0.20 | 0.20 | 0.40 | 0.40 | 0.80 | 0.80 |
| Vitamin A supplementation in infants and children 6-59 months | 2.90 | 2.90 | 2.90 | 2.90 | 2.90 | 2.90 | 2.90 | 5.80 | 5.80 | 11.60 | 11.60 |
| Pneumonia treatment (children) | 8.80 | 8.80 | 8.80 | 8.80 | 8.80 | 8.80 | 8.80 | 17.60 | 17.60 | 35.20 | 35.20 |
| Zinc | 1.00 | 1.00 | 1.00 | 1.00 | 1.00 | 1.00 | 1.00 | 2.00 | 2.00 | 4.00 | 4.00 |

Per patient costs are reported in Appendix 2 Table 5. Data from WHO-CHOICE (Data Appendix Table 1) are given in cost per year (I$, millions) per one million population and DALYs averted per year per one million population. In order to be able to discuss our results in the context of available estimates of the marginal productivity of country health care systems in the AFR-E region, we convert the costs from I$, millions to 2015 US$. Costs are first converted to local currency (using population weighted average of AFR-E region I$ to local currency conversion rates), then inflated from 2000 to 2015 (using population weighted average AFR-E region inflation rates), then converted to US$ (using population weighted average local currency to USD conversion rates) (Coinnews Media Group LLC, 2018; Kumaranayake, 2000; White, Conteh, Cibulskis, & Ghani, 2011).^[[2]](#footnote-2)^ This provides the cost in 2015 US$ for the AFR-E region reported in Appendix 2 Table 5 below.

| Appendix 2 Table 5. Per patient cost (2015 US$) (i.e. marginal increase in cost per patient) | | | | | | | | | | | | |
| --- | --- | --- | --- | --- | --- | --- | --- | --- | --- | --- | --- | --- |
| Intervention | 50% | 55% | 60% | 65% | 70% | 75% | 80% | 85% | 90% | 95% | 100% |  |
| Community newborn care package | 1.54 | 1.62 | 1.70 | 1.78 | 1.86 | 1.94 | 2.01 | 2.09 | 2.17 | 2.25 | 2.33 |  |
| Tetanus toxoid | 0.00 | 0.64 | 1.28 | 1.92 | 2.55 | 3.19 | 3.83 | 4.47 | 5.10 | 5.74 | 6.38 |  |
| Screening and treatment of syphilis | -0.17 | 0.26 | 0.70 | 1.13 | 1.57 | 2.00 | 2.44 | 2.87 | 3.31 | 3.74 | 4.18 |  |
| Normal delivery by a skilled attendant | 2.18 | 2.53 | 2.88 | 3.23 | 3.57 | 3.92 | 4.27 | 4.62 | 4.97 | 5.32 | 5.66 |  |
| Management of maternal sepsis | 0.71 | 1.16 | 1.62 | 2.08 | 2.53 | 2.99 | 3.45 | 3.90 | 4.36 | 4.81 | 5.27 |  |
| Management of serious newborn infections | 0.49 | 1.57 | 2.65 | 3.73 | 4.81 | 5.90 | 6.98 | 8.06 | 9.14 | 10.22 | 11.30 |  |
| Measles rubella vaccine | 0.65 | 1.09 | 1.53 | 1.97 | 2.41 | 2.84 | 3.28 | 3.72 | 4.16 | 4.60 | 5.03 |  |
| Insecticide-treated bed nets (ITN) | 4.91 | 4.91 | 4.91 | 4.91 | 4.91 | 4.91 | 4.91 | 4.91 | 4.91 | 4.91 | 4.91 |  |
| Intermittent presumptive treatment in pregnancy (IPTP) | 0.03 | 0.05 | 0.07 | 0.08 | 0.10 | 0.12 | 0.14 | 0.16 | 0.18 | 0.20 | 0.22 |  |
| Case management of malaria with artemisinin-based combination therapy (ACT) | 0.21 | 0.25 | 0.30 | 0.34 | 0.38 | 0.42 | 0.47 | 0.51 | 0.55 | 0.59 | 0.64 |  |
| Treatment of new smear-positive TB cases only under DOTS | 5.20 | 7.06 | 8.92 | 10.79 | 12.65 | 14.51 | 16.38 | 18.24 | 20.10 | 21.97 | 23.83 |  |
| Vitamin A supplementation in pregnant women | 2.83 | 5.25 | 7.68 | 10.10 | 12.52 | 14.95 | 17.37 | 19.80 | 22.22 | 24.64 | 27.07 |  |
| Management of severe malnutrition (children) | 95.01 | 95.61 | 96.21 | 96.81 | 97.42 | 98.02 | 98.62 | 99.22 | 99.83 | 100.43 | 101.03 |  |
| Vitamin A supplementation in infants and children 6-59 months | 45.64 | 48.07 | 50.49 | 52.91 | 55.34 | 57.76 | 60.18 | 62.61 | 65.03 | 67.45 | 69.88 |  |
| Pneumonia treatment (children) | 2.84 | 4.32 | 5.80 | 7.28 | 8.76 | 10.24 | 11.72 | 13.20 | 14.68 | 16.16 | 17.64 |  |
| Zinc | 0.77 | 0.85 | 0.93 | 1.01 | 1.09 | 1.17 | 1.25 | 1.33 | 1.41 | 1.49 | 1.57 |  |

The following three tables report incremental cost effectiveness ratios (ICERs). Because the cost functions (and sometimes also the benefit functions) for each intervention are typically non-linear, the marginal cost of increasing the breadth of coverage of an intervention from 50% to 55% may be lower than the marginal cost of increasing it from 85% to 90%. In these circumstances, the ICER of moving from 50% to 55% coverage would be lower than the ICER of moving from 85% to 90% coverage. The ICERs reported in Appendix 2 Tables 6-8 relate to steps 1 and 2 in the methods section of this paper. Note that because some intervention coverage alternatives in scenario 2 (for which ICERs are reported in Appendix 2 Table 7) are extendedly dominated (ED), step 3 requires recalculating the ICERs for this scenario after dropping any coverage levels that would be impossible to achieve with the remaining budget.

| Appendix 2 Table 6. Incremental Cost Effectiveness Ratios (ICERs) (Not Equity Weighted) (2015 US$) | | | | | | | | | | | | |
| --- | --- | --- | --- | --- | --- | --- | --- | --- | --- | --- | --- | --- |
| Intervention | 50% | 55% | 60% | 65% | 70% | 75% | 80% | 85% | 90% | 95% | 100% |  |
| Community newborn care package | 72 | 75 | 79 | 83 | 86 | 90 | 94 | 97 | 101 | 105 | 108 |  |
| Tetanus toxoid | 0 | 69 | 137 | 206 | 275 | 343 | 412 | 480 | 549 | 617 | 686 |  |
| Screening and treatment of syphilis | -191 | 293 | 776 | 1,260 | 1,743 | 2,226 | 2,710 | 3,193 | 3,677 | 4,160 | 4,644 |  |
| Normal delivery by a skilled attendant | 260 | 301 | 343 | 384 | 426 | 467 | 509 | 550 | 591 | 633 | 674 |  |
| Management of maternal sepsis | 393 | 646 | 900 | 1,153 | 1,407 | 1,660 | 1,914 | 2,168 | 2,421 | 2,675 | 2,928 |  |
| Management of serious newborn infections | 90 | 286 | 483 | 679 | 875 | 1,072 | 1,268 | 1,465 | 1,661 | 1,858 | 2,054 |  |
| Measles rubella vaccine | 131 | 229 | 337 | 457 | 590 | 739 | 907 | 1,097 | 1,316 | 1,569 | 1,865 |  |
| Insecticide-treated bed nets (ITN) | 246 | 255 | 265 | 276 | 288 | 300 | 314 | 330 | 347 | 366 | 387 |  |
| Intermittent presumptive treatment in pregnancy (IPTP) | 114 | 193 | 275 | 360 | 449 | 542 | 640 | 742 | 848 | 960 | 1,077 |  |
| Case management of malaria with artemisinin-based combination therapy (ACT) | 11 | 13 | 16 | 19 | 21 | 25 | 28 | 31 | 35 | 39 | 44 |  |
| Treatment of new smear-positive TB cases only under DOTS | 38 | 52 | 65 | 79 | 93 | 106 | 120 | 134 | 147 | 161 | 175 |  |
| Vitamin A supplementation in pregnant women | 976 | 1,812 | 2,647 | 3,483 | 4,319 | 5,154 | 5,990 | 6,826 | 7,662 | 8,497 | 9,333 |  |
| Management of severe malnutrition (children) | 475,033 | 478,046 | 481,058 | 484,071 | 487,083 | 490,096 | 493,108 | 496,121 | 499,134 | 502,146 | 505,159 |  |
| Vitamin A supplementation in infants and children 6-59 months | 15,739 | 16,574 | 17,410 | 18,246 | 19,082 | 19,917 | 20,753 | 21,589 | 22,424 | 23,260 | 24,096 |  |
| Pneumonia treatment (children) | 323 | 491 | 659 | 827 | 996 | 1,164 | 1,332 | 1,500 | 1,668 | 1,837 | 2,005 |  |
| Zinc | 772 | 852 | 932 | 1,012 | 1,092 | 1,171 | 1,251 | 1,331 | 1,411 | 1,491 | 1,570 |  |

| Appendix 2 Table 7. Equity Weighted Incremental Cost Effectiveness Ratios (ICERs) (2015 US$) | | | | | | | | |  |  |  |
| --- | --- | --- | --- | --- | --- | --- | --- | --- | --- | --- | --- |
| Intervention | 50% | 55% | 60% | 65% | 70% | 75% | 80% | 85% | 90% | 95% | 100% |
| Community newborn care package | ED | ED | ED | ED | ED | ED | ED | ED | ED | ED | 58 |
| Tetanus toxoid | 0 | 69 | 137 | 206 | ED | ED | ED | ED | ED | ED | 224 |
| Screening and treatment of syphilis | -191 | 293 | 776 | 1260 | ED | ED | ED | ED | ED | ED | 1490 |
| Normal delivery by a skilled attendant | 260 | ED | ED | ED | ED | ED | ED | ED | ED | ED | 271 |
| Management of maternal sepsis | 393 | 646 | 900 | ED | ED | ED | ED | ED | ED | ED | 1020 |
| Management of serious newborn infections | 90 | 286 | 483 | 679 | ED | ED | ED | ED | ED | ED | 684 |
| Measles rubella vaccine | 131 | 229 | 337 | 457 | ED | ED | ED | ED | ED | ED | 552 |
| Insecticide-treated bed nets (ITN) | ED | ED | ED | ED | ED | ED | ED | ED | ED | ED | 210 |
| Intermittent presumptive treatment in pregnancy (IPTP) | 114 | 193 | 275 | ED | ED | ED | ED | ED | ED | ED | 355 |
| Case management of malaria with artemisinin-based combination therapy (ACT) | 11 | 13 | ED | ED | ED | ED | ED | ED | ED | ED | 15 |
| Treatment of new smear-positive TB cases only under DOTS | 38 | 52 | ED | ED | ED | ED | ED | ED | ED | ED | 64 |
| Vitamin A supplementation in pregnant women | 976 | 1812 | 2647 | ED | ED | ED | ED | ED | ED | ED | 3204 |
| Management of severe malnutrition (children) | ED | ED | ED | ED | ED | ED | ED | ED | ED | ED | 345227 |
| Vitamin A supplementation in infants and children 6-59 months | ED | ED | ED | ED | ED | ED | ED | ED | ED | ED | 12883 |
| Pneumonia treatment (children) | 323 | 491 | 659 | ED | ED | ED | ED | ED | ED | ED | 708 |
| Zinc | ED | ED | ED | ED | ED | ED | ED | ED | ED | ED | 708 |

| Appendix 2 Table 8. Incremental Cost Effectiveness Ratios (ICERs) for 95% coverage (2015 US$) | |
| --- | --- |
| Intervention | 95% |
| Community newborn care package | 80 |
| Tetanus toxoid | 163 |
| Screening and treatment of syphilis | 954 |
| Normal delivery by a skilled attendant | 358 |
| Management of maternal sepsis | 993 |
| Management of serious newborn infections | 555 |
| Measles rubella vaccine | 380 |
| Insecticide-treated bed nets (ITN) | 269 |
| Intermittent presumptive treatment in pregnancy (IPTP) | 306 |
| Case management of malaria with artemisinin-based combination therapy (ACT) | 17 |
| Treatment of new smear-positive TB cases only under DOTS | 70 |
| Vitamin A supplementation in pregnant women | 2,955 |
| Management of severe malnutrition (children) | 482,168 |
| Vitamin A supplementation in infants and children 6-59 months | 17,718 |
| Pneumonia treatment (children) | 721 |
| Zinc | 961 |

1. One potential exception is through the quality of the provision of healthcare, but there is little available evidence that could be incorporated. [↑](#footnote-ref-1)
2. Population weights: World Bank (<https://data.worldbank.org/indicator/SP.POP.TOTL>) accessed 03/02/2019); I$ to local currency conversion rates: World Bank (<https://data.worldbank.org/indicator/PA.NUS.PPP>) accessed 03/02/2019; inflation rates: average of rates published by the IMF and World Bank as neither source has rates for all countries in the region (<https://www.imf.org/external/datamapper/PCPIPCH@WEO/BWA/BDI/CAF/COG/CIV/COD/ERI/SWZ/ETH/KEN/LSO/MWI/MOZ/NAM/RWA/ZAF/TZA/UGA/ZMB/ZWE> and <https://data.worldbank.org/indicator/FP.CPI.TOTL.ZG?locations=AE>) accessed 01/02/2019; local currency to USD conversion rates: Oanda.com accessed 03/02/2019 [↑](#footnote-ref-2)
